# Correction: The impact of a routine late third trimester growth scan on the incidence, diagnosis, and management of breech presentation in Oxfordshire, UK: A cohort study

**DOI:** 10.1371/journal.pmed.1003613

**Published:** 2021-04-22

**Authors:** Ibtisam Salim, Eleonora Staines-Urias, Sam Mathewlynn, Lior Drukker, Manu Vatish, Lawrence Impey

There is an error in the Author summary section in the first bullet point under the heading "What did the researchers do and find?" The correct sentence should read: "This study assessed breech outcomes of 27,825 term births before and after the introduction of a third trimester screening ultrasound, which increased the proportion of pregnancies screened from 39% to 99%".
