# Supplementary material for: The impact of a routine late third trimester growth scan on the incidence, diagnosis, and management of breech presentation in Oxfordshire, UK: A cohort study
Source: PLoS Med. 2021 Jan 15;18(1):e1003503. doi: 10.1371/journal.pmed.1003503 (PMC7810318; doi:10.1371/journal.pmed.1003503)
Supplement: S1 Analysis plan — (DOCX) [file pmed.1003503.s002.docx]

**The impact of a routine 36 week growth scan on the diagnosis and management of breech presentation.**

**Salim I, Impey L.**

**Aim:**

To determine the impact of a routine third trimester ultrasound scan on babies with a breech presentation at term

**Methods:**

Impact study of prospectively collected data

Population under study: all singleton, non-anomalous babies who had both a 20 week scan and who delivered from 35+0 weeks, divided according to whether their EDD was …when a 36 week scan was introduced for all women: (EDD Oct 2015- Sept 2016 (pre) and EDD Oct 2016-Sept 2017)

Data: EPR, ViewPoint & Badgerrnet

Analysis: Stata

Ethics: OxGRIP; funding: none

**Results**

Primary outcomes: compare 1) Overall incidence of breech presentation 2) Incidence of undiagnosed breech presentation.

Secondary outcomes: 1) ECV clinic outcomes 2) Mode of birth of breech presentation 3) Perinatal outcome

**Table 1: Demographics of population**

Pre- 36 week scan Post 36 week scan RR (95%CI) / p= N (%) N(%)

Total cohort (scan at 20w)

Total del >36 w in OUH

Age

BMI

Parity=0

Caucasian race

Birthweight

Had a scan >35+0 weeks

**Table 2: ECV outcomes**

Pre- 36 week scan Post 36 week scan RR (95%CI) / p=

N (%) N(%)

Total del >36 w in OUH

Total in breech clinic*

Total breech

ECV declined

ECV attempted

ECV successful

**NB Remember to exclude tertiary referrals for breech only*

**Table 3: Delivery outcomes of all breech** **babies**

Pre- 36 week scan Post 36 week scan RR (95%CI) / p=

N (%) N(%)

Total del >36 w in OUH

Breech at delivery

Gestation at del

Planned VBB

Actual VBB

Planned CS

Actual VBB

Undiagnosed*

Actual VBB

*Defined as breech at del 1) without breech at any scan > 35+0 weeks or 2) ECV successful (may need to look at the notes of ones who had no scan >35+0)

**Table 4: Perinatal outcomes of breech babies**

Pre- 36 week scan Post 36 week scan RR (95%CI) / p=

N (%) N(%)

Breech at delivery

? Maternal:

Em CS

Perinatal:

Mortality

NNU admission

NCAO

Apgar <7 at 5 min

BW <5^th^ c

Gestation <37 w

**Flow diagram of outcomes in women in post 36 week scan period: this may be useful simply for ensuring we do not miss crucial step in our data section and analysis.**

Women in cohort

Presentation at 36 week scan del/ moved away no scan

Breech Cephalic Other

ECV attempted ECV not attempted

ECV success ECV fail

Breech at delivery Cephalic at del Other at del

Planned VBB Planned CS* Attempted VBB

Successful VBB

*Cs planned irrespective of whether done as em or el procedure

**Define the dataset:**

1. Singleton, had 20 week scan in OUH, no fetal anomaly (probably not just detected antenatally because these may be difficult to differentiate from those detected afterwards although I think we have a code). *First number(for table 1).*
2. Then: delivered in OUH from 35+0 weeks (i.e. outcome data). *Second number (tables 2-4).* Also declare number delivered in OUH between 20 and 34+6 weeks, and subtract latter from former to give number delivered elsewhere.
3. Then: subset 1 for table 2: seen in breech clinic. Even if cephalic. You must have this data from what you have already done. But we may need to amalgamate this list into main database
4. Then: subset 2 for Table 3 and 4: breech at delivery.
