# Supplementary material for: The impact of a routine late third trimester growth scan on the incidence, diagnosis, and management of breech presentation in Oxfordshire, UK: A cohort study
Source: PLoS Med. 2021 Jan 15;18(1):e1003503. doi: 10.1371/journal.pmed.1003503 (PMC7810318; doi:10.1371/journal.pmed.1003503)
Supplement: Attachment — Submitted filename: PLOSMed_RoutineUSBreech.docx [file pmed.1003503.s004.docx]

PLOS Medicine Breech paper review – to authors

General and specific comments included below. This paper is highly topical to an obstetric/maternity care audience, as the role of routine third trimester ultrasound continues to be considered in many developed settings worldwide.

There are already a few major papers looking at the identification of breech babies via routine T3 US and the consequent impact on care/outcomes – this paper attempts to investigate whether routine T3 ultrasound results in changes in antenatal detection, mode of birth and incidence of breech presentation in a ‘real world’ setting (primips and multips).

I think the premise is good and an important question but I think the findings that form the focus of this paper are not those that are most clinically relevant or significant. The paper concludes that routine T3 US did not reduce the presentation of breech at term but for a clinical audience, it is much more interesting and salient that routine US *did* significantly reduce the incidence/risk of undiagnosed breech before birth and labour – this is where the majority of breech-related morbidity lies so it would seem to be of great significance but is not how this paper is framed. I would suggest reframing the discussion of this paper if it is intended to be published for a clinical audience.

Abstract

In the conclusion, an erroneous ‘of’

Intro

States CS is ‘more costly’ – than what? Vaginal birth? Because elective CS for breech is arguably much less costly than breech vaginal birth. This needs to be clarified.

The paper states that the greatest negative of breech presentation at term is the contribution to CS rate but the key end-point/question of the study is incidence of breech presentation at term. Breech at term doesn’t really matter in the clinical setting unless it changes a clinical outcome, such as emergency CS rate for undiagnosed breech specifically or unplanned vaginal breech births.

Methods

Only one line given about determining RR – how was this done statistically? This would seem a good study in which to use causal inference methodology and attempt to replicate the assumptions of an RCT, especially given the clear exposure of universal US and known differences in the demographic characteristics of pre and post US cohorts.

Results

Summary of key findings:

No change in term breech presentation (S)

More breech presentation at clinic (S)

More planned breech VD (NS)

Fewer actual breech VD (S)

More elective CS overall (S)

Fewer emergency CS overall (NS)

**Significant reduction in undiagnosed breech before birth and before labour** – these are narrow CI and surely clinically significant findings!

Hard to work out how (Table 1) gestational age at delivery could be significantly different between the pre and post-OxGRIP groups given numbers provided?

Table 1 reports breech at delivery as 2.6 vs 2.7 and the text states breech at term is the same – these are potentially different things, needs to be clarified if being used interchangeably.

Planned vaginal breech increased but actual vaginal breech reduced overall (not in those in whom breech was diagnosed) – Was this because X% of planned breech births reverted to cephalic before birth or because they were delivered by CS??

Table 3 indicates that undiagnosed breech before birth was significantly reduced from 25.5% to 8.3% and that amongst the undiagnosed breech babes at term, breech undiagnosed before labour was significantly reduced – this seems to be an important clinical end-point.

Error – ‘the proportion of breech babies giving birth’ does not make sense – ‘being born vaginally’ perhaps?

Also, ‘a surrogate for babies that were breech in the third trimester babies’ – an extra ‘babies’?

It would be informative to know whether those two breech babies that died pre-OxGRIP were born via vaginal birth or were undiagnosed breech even?

The study is likely under-powered to detect any meaningful reductions in adverse perinatal outcomes but still demonstrates a trend towards reduction across all outcomes – seems important in the context of the study.

Discussion

The authors seem to emphasise the findings of the study that are not of greatest clinical significance. The steady rate of term breech presentation pre and post routine T3 US is not that interesting, but the significant reduction in actual vaginal breech births and in undiagnosed breech presentation at birth or pre-labour is of great clinical significance and will get the attention of a clinical readership – would seem to justify the intervention. Further, although not powered to determine significance, the trend towards better neonatal outcomes, though rare, is also meaningful for clinicians. This is the *main* reason to care about breech presentation at term and therefore the role of routine T3 US, not so much the change in breech presentation or caesarean section rates.

Error: First paragraph says ‘at last’ instead of ‘at least’

Error: ‘Whilst the gestation at the time of the ECV gestation at ECV’ – sentence does not make sense
