# Supplementary material for: The impact of a routine late third trimester growth scan on the incidence, diagnosis, and management of breech presentation in Oxfordshire, UK: A cohort study
Source: PLoS Med. 2021 Jan 15;18(1):e1003503. doi: 10.1371/journal.pmed.1003503 (PMC7810318; doi:10.1371/journal.pmed.1003503)
Supplement: Attachment — Submitted filename: Response to reviewer_R1.docx [file pmed.1003503.s005.docx]

17^th^ September 2020

The Editors

PLOS Medicine

Carlyle House

Carlyle Road

Cambridge, CB4 3DN

United Kingdom

Dear Editors,

**RE: Manuscript ID PMEDICINE-D-20-00199R1 entitled “The impact of a routine late third trimester growth scan on the incidence, diagnosis and management of breech presentation in Oxford, UK: A cohort study”**

Thank you very much for returning these reviews to us and for considering our paper. We apologise profusely for the need for most of the editorial comments and for the small delay in returning this to you.

Responding to the reviews has made the paper very much clearer. We very much hope you will accept this for publication in your journal.

Yours Sincerely

Ibtisam Salim (Corresponding Author) & Lawrence Impey

on behalf of the coauthors.

Dr Ibtisam Salim MD, MMED (Obs/Gyn), MRCOG

Nuffield Department of Women’s and Reproductive Health

Level 3, Womens Centre

John Radcliffe Hospital

Headley Way, Headington

Oxford

OX3 9DU

Email: ibtisam.salim@wrh.ox.ac.uk

Tel: +441865 221004

**Editorial comments:**

1.You mention that all relevant data are included - are you able include patient-level data in supplementary files, for example?

*Authors Response: Yes, if requested. We have not included this with the resubmission but certainly can do so. Please note that even with only the variables of interest to this actual paper, this is a vast file.*

2. Please quote the setting in the title (e.g., "in Oxford, UK") and add a study descriptor (e.g., "a cohort study") following a colon.

*Authors Response: This has been done. Now reads: The impact of a routine late third trimester growth scan on the incidence, diagnosis and management of breech presentation in Oxford, UK: A cohort study*

3.Please restructure the abstract to generate a combined "Methods and findings" section according to journal style. The final sentence of the new combined subsection should quote 2-3 of the study's main limitations.

*Authors Response: This has been added in the abstract. (Page 2, lines 25-42)*

4.In your abstract, please quote aggregate demographic details for study participants.

*Authors Response: This has been done. (Page 2)*

5.After the abstract, please add a new and accessible "Author summary" section in non-identical prose. You may find it helpful to consult one or two recent research papers published in PLOS Medicine to get a sense of the preferred style.

*Authors Response: This has been added. (Page 4, lines 49-78)*

6.Early in the methods section of your main text, please state whether or not the study had a protocol or prespecified analysis plan (and if so attach the relevant document(s) as supplementary file(s)). Please highlight analyses that were not prespecified.

*Authors Response: We have stated that these pre specified analyses early in the methods as requested. (Page 7-10, line 123-125)*

*We did not register a pre-specified analysis plan, because this was not a randomized controlled trial but an analysis of whether a service initiative led to changes for which we had aimed in introducing it. However we enclose dated evidence that we planned the study before analysing the data (Others:Prespecified Analysis). I have opened but not altered the file and so its ‘last modified date’ should stand. We are not sure if you will want to publish this document as a supplementary file* because the study was not pre registered, it was not a trial and because the document itself is quite messy.

7.We notice an ethics approval date in 2017 quoted in the methods section - please explain how this relates to the date the study started. Also, we suggest noting the situation regarding participant informed consent - perhaps not required in routine care.

*Authors Response: The introduction of the routine ultrasound scan was a clinical service change. Routine patient-level data were, as before, prospectively collected in several datasets: Cerner, Viewpoint and Badgernet. Following ethical approval, these were merged, cleaned and analysed. Ethical approval was for generic analysis and not specifically related to this analysis. Informed patient consent was not required. (Page 10, line 181-183)*

8. In table 1, we suggest "White" rather than "Caucasian" ethnicity.

*Authors Response: This has been done. Please note we corrected an error in the % of white ethnicity in the ‘pre’ group. The rest of this table has been re checked. (Page 11, line 193)*

9. Please adapt the first paragraph of your "Discussion" section to provide a summary of the study findings - i.e., employing wording such as "we found that ... was" rather than the more general "shows this is not the case", for example, with the latter moved to a subsequent paragraph if you wish.

*Authors Response: This has been done. (Page 16, line 248-257)*

10.In the "Conclusion" section at the end of the main text, please adapt the text to "In this study, we found that ... did not lead ..." to avoid over-generalizing conclusions from a single-centre study.

*Authors Response: This has been done. (Page 19, line 325-329)*

11.Please quote p values alongside 95% CI throughout the paper, where available.

*Authors Response: This has been done.*

12.Throughout the text, please format reference call-outs as follows: "... preterm births [1].".

*Authors Response: This has been done*

13.Please remove all iterations of "[Internet]" from the reference list.

*Authors Response: This has been done. (Page 20, line 348- 392)*

14.Please add a completed checklist for the most appropriate reporting guideline, which we suspect will be STROBE, as a supplementary document (referred to in the methods section, e.g., "See S1_STROBE_Checklist"). In the checklist please refer to individual items by section (e.g., "Methods") and paragraph number rather than by line or page numbers, as the latter generally change in the event of publication.

*Authors Response: This has been done. See S1_STROBE checklist.*

----------------------------------------------------------------------------------------------

Comments from the reviewers:

**Reviewer #1:**

This article is about the impact that a change in procedure to routinely have late trimester scans has on various outcomes including breech presentation.

As a statistical reviewer, there is very little statistical considerations being reported in the article for me to be able to comment on, and only basic summary statistics and unadjusted two-sample tests are used to compare between the populations before and after the change in procedure. Similarly, given the methods of data collection there is very little on study design or methods that is relevant to comment on. Perhaps the only comment I would have for the statistics, is that the method of calculation for the Risk Ratio confidence interval should be stated.

*Authors Response: This has been done. Page 8, line 139- 142*

The above comments are not implying that the article is not of value or that further statistics/analysis would be needed to answer the key question of interest, just that there are only very basic numbers reported and so the value of the paper will almost entirely be based on clinical judgement and relevance for which I couldn't reasonably contribute towards.

General and specific comments included below.

1.This paper is highly topical to an obstetric/maternity care audience, as the role of routine third trimester ultrasound continues to be considered in many developed settings worldwide.

*Authors Response: Thank you.*

2.There are already a few major papers looking at the identification of breech babies via routine T3 US and the consequent impact on care/outcomes - this paper attempts to investigate whether routine T3 ultrasound results in changes in antenatal detection, mode of birth and incidence of breech presentation in a 'real world' setting (primips and multips).

I think the premise is good and an important question but I think the findings that form the focus of this paper are not those that are most clinically relevant or significant. The paper concludes that routine T3 US did not reduce the presentation of breech at term but for a clinical audience, it is much more interesting and salient that routine US did significantly reduce the incidence/risk of undiagnosed breech before birth and labour - this is where the majority of breech-related morbidity lies so it would seem to be of great significance but is not how this paper is framed. I would suggest reframing the discussion of this paper if it is intended to be published for a clinical audience.

*Authors Response: We have re-ordered the abstract, introduction and discussion to give equal emphasis to undiagnosed breech and the overall incidence of breech. Nevertheless, we wish to justify the importance of the overall incidence and our (surprising) findings. It was reasonable to believe that a reduction in breech presentation would occur through increased ECV as a result of increased antenatal detection, and this would have been an expected and major benefit. Breech presentation is associated with caesarean section, the worldwide rates of which are considered by many, including the WHO, to be much too high. It is also associated with increased morbidity and mortality at vaginal birth. It is therefore extremely important to show that the introduction of a third trimester scan does not reduce the incidence of breech presentation, particularly in a unit with widespread and successful use of ECV.*

Abstract

3.In the conclusion, an erroneous 'of'

*Authors Response: Thank you: this has been removed. (Page 3, line 46)*

Intro

4.States CS is 'more costly' - than what? Vaginal birth? Because elective CS for breech is arguably much less costly than breech vaginal birth. This needs to be clarified.

*Authors Response: This has been altered. The comment pertains to the potential benefit (see answer to Reviewer 2, point 2). (Page 6, lines 85-88)*

5.The paper states that the greatest negative of breech presentation at term is the contribution to CS rate but the key end-point/question of the study is incidence of breech presentation at term. Breech at term doesn't really matter in the clinical setting unless it changes a clinical outcome, such as emergency CS rate for undiagnosed breech specifically or unplanned vaginal breech births.

*Authors Response: Thank you. The 2 end points are the incidence of breech presentation and the incidence of undiagnosed breech presentation. Breech presentation inevitably alters a clinical outcome: either a caesarean section, or a vaginal breech birth. See response to Reviewer 2, point 2.*

Methods

6.Only one line given about determining RR - how was this done statistically? This would seem a good study in which to use causal inference methodology and attempt to replicate the assumptions of an RCT, especially given the clear exposure of universal US and known differences in the demographic characteristics of pre and post US cohorts.

*Authors Response: Thank you. Please see response to reviewer 1, point 1*

*The comment regarding causal inference methodology is welcome. We debated whether to use adjusted RRs for exploratory analysis, but none of the minor alterations in demographics could meaningfully be associated with breech presentation or a failure to diagnose it. Table 1 demonstrates the principal alterations during the study period, and the alteration in the use of third trimester ultrasound is by far the greatest.*

Results

7.Summary of key findings:

No change in term breech presentation (S) More breech presentation at clinic (S) More planned breech VD (NS) Fewer actual breech VD (S) More elective CS overall (S) Fewer emergency CS overall (NS) Significant reduction in undiagnosed breech before birth and before labour - these are narrow CI and surely clinically significant findings!

Hard to work out how (Table 1) gestational age at delivery could be significantly different between the pre and post-OxGRIP groups given numbers provided?

*Authors Response: Thank you. This was an error and has been rectified. See response to editor comments point 8. The rest of this table has been re checked. (Page 11, line 193 Table 1)*

8.Table 1 reports breech at delivery as 2.6 vs 2.7 and the text states breech at term is the same - these are potentially different things, needs to be clarified if being used interchangeably.

*Authors Response: Thank you. This has been rectified (Page 11, lines 197-8)*

9.Planned vaginal breech increased but actual vaginal breech reduced overall (not in those in whom breech was diagnosed) - Was this because X% of planned breech births reverted to cephalic before birth or because they were delivered by CS??

*Authors Response: It was because there were many fewer unplanned vaginal breech births because the breeches were not undiagnosed. This has been better explained in the text. (Page 16, lines 237-241)*

10.Table 3 indicates that undiagnosed breech before birth was significantly reduced from 25.5% to 8.3% and that amongst the undiagnosed breech babes at term, breech undiagnosed before labour was significantly reduced - this seems to be an important clinical end-point.

*Authors Response: Thank you. We agree: this is why we present it this way, allowing exclusion of those that were not known prior to an elective caesarean section, where the presentation matters far less.*

11.Error - 'the proportion of breech babies giving birth' does not make sense - 'being born vaginally' perhaps?

*Authors Response: This has been amended. (Page 14, line 214)*

12.Also, 'a surrogate for babies that were breech in the third trimester babies' - an extra 'babies'?

*Authors Response: Thank you. The extra ‘babies’ has been removed. (Page 15, lines 224-226)*

13.It would be informative to know whether those two breech babies that died pre-OxGRIP were born via vaginal birth or were undiagnosed breech even?

*Authors Response: Thank you. This is now done. (Page 15, lines 226-230)*

14.The study is likely under-powered to detect any meaningful reductions in adverse perinatal outcomes but still demonstrates a trend towards reduction across all outcomes - seems important in the context of the study.

*Authors Response: Thank you. We have amended the text. (Page 15, line 226-228)*

Discussion

15.The authors seem to emphasise the findings of the study that are not of greatest clinical significance. The steady rate of term breech presentation pre and post routine T3 US is not that interesting, but the significant reduction in actual vaginal breech births and in undiagnosed breech presentation at birth or pre-labour is of great clinical significance and will get the attention of a clinical readership - would seem to justify the intervention. Further, although not powered to determine significance, the trend towards better neonatal outcomes, though rare, is also meaningful for clinicians. This is the main reason to care about breech presentation at term and therefore the role of routine T3 US, not so much the change in breech presentation or caesarean section rates.

*Authors Response: This has been addressed. Please see response to point 2.*

16. Error: First paragraph says 'at last' instead of 'at least'

*Authors Response: Thank you. This has been amended. (Page 16, Line 247)*

17. Error: 'Whilst the gestation at the time of the ECV gestation at ECV' - sentence does not make sense

*Authors Response: Thank you. This has been amended. (Page18, lines 297-300)*

**Reviewer #3:**

General comments:

1. This study describes the ‘real obstetrical practice’ after implementing a routine scan near term (after 35 weeks gestational age) to detect growth restricted fetus and as an additional finding, fetal positioning. This all is situated in a perinatal center with a specialized clinic for external cephalic version (ECV). The numbers of attempted ECV was high (87.3%) with a success rate of 44.3%. Routine implication of this ultrasound scan did result in 5 times more often detected breech presentation as was one of the main outcomes. However, the incidence of breech presentation at delivery was equal (2.6% v 2.7%) to the historical cohort not exposed to a routine ultrasound scan In addition no differences were shown in relevant outcomes like neonatal condition, emergency CS or others. despite of the fact that undiagnosed breech position was reduced considerably. The comparison of historical cohorts was the best available since near term ultrasound was implemented in daily practice for other reasons than identifying breech position and probably this holds for many centers worldwide. This means that not finding impact on outcome of the obstetrical clinical situation of breech at term probably will not change the use of ultrasound at term in daily practice and therefore detecting non-cephalic position

*Authors Response: Thank you. We believe the study to be more than a comparison with a historical cohort. In assessing all term women before and after, with data demonstrating the increase in ultrasound, we are able to assess the real world effect of such a change in a large maternity unit*

2. The authors argue that even in a setting where vaginal birth is actively discouraged, maintaining clinical skills for management of vaginal breech birth remains important. The importance of the study therefore lies in the fact that it shows how many women will deliver a breech child vaginally requiring unexpected specific obstetrical skills of the attending physician or midwife. In this cohort of more than 13,000 women there are 2 and neonatal outcome of the two deliveries is not reported.

*Authors Response: Thank you. We now acknowledge this better in the text. Please note there were still 17 actual vaginal breech births in the planned vaginal birth group. Both the 2 undiagnosed breech babies were born in good condition. (Page17, Line 258-266)*

3. The study is performed well and the data are discussed clearly and thoroughly. Strengths and weaknesses are discussed adequately. However diagnostic procedures though allowing for more informed choices also result in unnecessary cost and stress in women in whom spontaneous correction to cephalic presentation occurs. Furthermore, if diagnosing by term ultrasound is important one would expect that most adverse perinatal outcome is found in the women with undiagnosed breech in both cohorts. Since the historical cohorts do not differ in perinatal outcome this might be important to discuss. Are there any data on how women experienced care?

*Authors Response: Thank you. Unfortunately, patient level data on women’s experiences is not routinely collected. As noted by the previous reviewer, even the numbers presented are probably inadequate to detect differences in severe adverse neonatal outcomes. We do now discuss the perinatal outcome data.*

4. Concluding: it is not clear whether this policy changes maternal or perinatal outcome and the use of near term ultrasound probably will continue. Though it answers some of the questions posed in the introduction it does not change practice and results are of importance for obstetrics and midwifery.

*Authors Response: Thank you. We agree. We do however note that the use of third trimester universal ultrasound to detect breech presentation is currently under scrutiny by the National Screening Committee in the UK and this study will provide valuable evidence.*

Specific comments:

Introduction:

5. We would suggest also to discuss the clinical dilemma of breech delivery from a perspective of mothers choosing vaginal breech delivery.

*Authors Response: Thank you. We have done this. (Page 8, lines 150- 153)*

6. Please be consistent in the use of Caesarean section, CS and caesarean.birth

*Authors Response: Thank you. We have done this, using CS, although left the word caesarean in the author summary and abstract.*

Methods:

7. In the second paragraph, the ECV procedure is described very briefly. “Ultrasound is performed in the clinic and women are assessed for their suitability for ECV which, if appropriate and accepted, is performed in the same clinic.” What does suitability mean? What are the documented contra-indications, when is a woman ‘appropriate’ for ECV, who performs ECV and in what setting? Is routine uterine relaxation with a beta-mimetic available? Does epidural analgesia for this matter exist in your clinic? The success rate is very high, so therefore, I believe there is an optimal setting. However, this is not clear from this manuscript. Did the procedure change during the period the cohort was studied?

*Authors Response: Thank you. In the interest of brevity, we did not describe the procedure in detail. We now reference the UK national guidelines and our own published ECV data here and hope this is sufficient. The procedure did not change at all and we have added this. (Page 8, lines 146-153)*

8. Further: “In persistent breech presentations, the mode of birth is discussed: women are assessed for their suitability for vaginal breech birth, which is planned depending on maternal request. Alternatively, elective Caesarean birth is offered.”

What does the assessment of women mean? Do you do pelvimetry? What are your contra-indications for a planned vaginal breech delivery (VBD)? Also, of importance what is your local protocol on assisting VBD. Who supervises VBD? Does simulation-based team training take place? Is induction for obstetrical reasons allowed? Is augmentation allowed (therapeutic or maybe prophylactic)? Is upright/all fours position during active phase optional for women? When did elective CD take place?

*Authors Response: Thank you. In the interests of brevity, we did not describe the circumstances of vaginal breech birth. We have now added a little more detail and provided an appropriate reference. (Page 8, line146-153)*

9. In the third paragraph, type of breech is not documented.

*Authors Response: The type of breech was not recorded in hospital dataset, although it is in our prospectively collected data on both ECV and vaginal breech birth. This means we were unable to provide complete data, but this does not affect our outcome measures.*

10. In the fourth paragraph, not all outcomes reported in the results section are mentioned. Therefore, as a reader, the results reported later seem to appear from nowhere. This paragraph needs revision. Please mention all important outcomes which are reported later. What are the primary and secondary outcomes? Maternal outcomes are not reported; why not, and please discuss this in the discussion section.

*Authors Response: Thank you. This has been done. The methods section has now the specified outcomes. See response to editor, point 6.*

*We have not presented maternal outcomes: this was because current hospital data collection on anything but blood loss is poor. We are currently trying to extract better data that would include readmission rates, although this would require yet another table. We accept and apologise for this failing and have added it to the limitations. (Page 19,line 315-317)*

Results:

11. I suggest to make a figure to demonstrate a flow chart of the Pre-OxGRIP cohort and of the Post-OxGRIP cohort. This will generate an overview for readers. Please report this first, then the table with all baseline characteristics. To increase readability further, please prespecify all used cohorts in the method section as multiple cohorts are been used by the authors. There is the whole cohort, the ‘referred to the breech clinic’ cohort, the ‘breech at delivery’ cohort and the ‘third trimester breech presentation’ cohort to report on neonatal outcome. The use of the same cohorts is advisable as the denominator changes constantly in the way it is reported now.

*Authors Response: Thank you for this suggestion.*

*Please note that not all the information is available in the Tables and 2 flow diagrams would be required- for both pre and post cohorts. As what appears to require clarification are the definitions of cohorts we have, in the interests of readability, understanding and simplicity, created a generic ‘possibilities flow’ with all the cohorts eg ‘undiagnosed breech’ clearly labelled to show how they were calculated*

*Please note also the difficulty with examining perinatal outcomes. This we felt was best reported on babies that were breech in the third trimester. This surrogate definition was used to assess the impact of the universal scan policy on all breeches in the third trimester (as opposed to those at delivery) to prevent bias due to better antenatal diagnosis, and exclusions of the potential detrimental effects of ECV. Please note that this cohort could be removed and the analysis performed only on breech presentation at term. This would be a simpler but in our view less comprehensive analysis. We will be guided by an editorial decision.*

12.The prespecified primary outcome is not reported in the method section, assuming this is breech presentation at delivery. This outcome is reported in table 1, a table to demonstrate demographic characteristics. In my opinion, this is not the correct manner. It can be considered to demonstrate only characteristics of ‘breech at delivery in the Pre-OxGRIP cohort’ and ‘breech at delivery in the Post-OxGRIP cohort’.

*Authors Response: Thank you. We absolutely agree. This has been amended as suggested and included in Table 2. Please note the Table 2 and 3 have been changed around to enable better presentation of the results and in response to this and Reviewer 3, point 13. (Page 13, Line 208)*

13. In table 3, actual vaginal delivery breech birth is described for the first time, does this mean successful vaginal breech birth? Also, I would suggest to distinguish mode of delivery into elective CS or planned VBD, and planned VBD is further specified in successful VBD (spontaneous and breech extraction) or emergency CS (for no progress of labor in first or second stage, suspected fetal distress and fetal position not compliant for VBD during planned VBD).

Also, in table 3, first the whole group is described (n=380 and n=360), after this, there is a division between ‘diagnosed’ and ‘undiagnosed’ breech presentation. Please use the same outcome as described above.

*Authors Response: Thank you. We have altered Table 3. It is unfortunately more complex than the reviewer suggests, as emergency CS could be for an undiagnosed breech, or for a failed attempt at planned, or unplanned vaginal breech birth. We hope this is made clearer in former Table 3 (now Table 2) and by the flow diagram. (Page 13-14, Page 208-210)*

14. In the third paragraph, preterm breech delivery is mentioned for this first time and a reference is made to table 4 (“There was no significant difference in the proportion of preterm births that were breech (Table 4).”). However, table 4 reports on neonatal outcome, is there a table lacking on preterm breech?

*Authors Response: Thank you for pointing out this mistake. An analysis of preterm breeches was originally included as a Table 5 to demonstrate that preterm breeches contributed considerably to overall breech presentation, and we did not adequately alter the text when we removed it. Now we have simply left it as a sentence in the results. (Page 10, lines 189-190)*

15. Neonatal outcome is not prespecified in the method section. Also, I’m missing maternal outcomes (already pointed out earlier).

*Authors Response: Thank you. The methods section has now the specified outcomes. Please see response to editor, point 6 and reviewer 3, point 10. (Page 7, lines 123-125 & Page 19, lines 315-317)*

16. In the second paragraph, failure rate is reported instead of success rate. Please report on success rate or failure rate consistently.

*Authors Response: Thank you. This been changed. (Page 12, line 204-206)*

Discussion

17. The authors start with presentation of two main findings, the reduction of undetected breech AND the reduction in term breech and continue with the statement that this is not the case. We would suggest starting with your primary outcome.

*Authors Response: Thank you. This has been amended. (Page 16, line 235-243)*

18. Further: “This is reflected in our estimation (Table 5) of the number of babies who were ‘probably breech’ at 36 weeks. This does not mean that ECV is not effective: meta-analysis of randomised controlled trials has shown it to reduce the number of breech presentations at birth (8). It is merely that a proportion of breech babies turn anyway (13).

*Authors Response: Thank you. We have removed the first (above) sentence as it is unnecessary. We are trying to explain that whilst there was an apparent increase in the number of successful ECVs, there were not fewer breech presentations at term. This was a surprise and a key finding of the study. (Page16, lines 241-242)*

19. First, I could be wrong but table 5 seems missing in the manuscript. To better understand this statement by the authors, a flow chart could be helpful. Please note that the number needed to treat for ECV to prevent 1 CD is 2.6 (95% CI 2.0 to 3.9) (source: De Hundt et al, Obs&Gyn, June 2014; 123(6):1327-34). Please clarify this remark.

*Authors Response: Thank you. Please see response to reviewer 3, point 14.*

19. “The numbers of preterm breeches demonstrate that these account for a substantial proportion (22.5%) of all breech presentations, and that it is in this group where most (54.3%) of vaginal breech births now occur.”

These numbers are not stated earlier. In the first paragraph of the result section, the vaginal delivery rate is reported to be 26.3%. Please explain this more carefully. The preterm breech delivery is still a topic of international discussion and in which the preferred mode of delivery is not known (yet).

*Authors Response: Thank you and we apologise for this. Please see response to reviewer 3, point 14.*

20. “The finding that term breech presentation is not reduced may be because of the relatively low existing incidence (2.6%) and is therefore only applicable to units with a comprehensive and successful ECV service.”

This is very good statement of the authors and needs to be spread more widely. Perhaps because of their well-functioning ‘Breech Clinic’ the low incidence already exists. The authors may state that a well-functioning ‘ECV Clinic’ is needed in hospitals and that if not, eligible patients need to be referred.

*Authors Response: Thank you. We have considerably amended the discussion to take account of this. (Pages 18-19, lines 303-309)*

21. The general number of women opting for a planned VBD is low in your population compared to other countries like Sweden, Denmark or The Netherlands. Please discuss this since it could be that in those countries the impact of term scans could be different for term breech delivery.

*Authors Response: Thank you: we agree. This has been added to the discussion. (Page 19, lines 314-315)*
