# Supplementary material for: The impact of a routine late third trimester growth scan on the incidence, diagnosis, and management of breech presentation in Oxfordshire, UK: A cohort study
Source: PLoS Med. 2021 Jan 15;18(1):e1003503. doi: 10.1371/journal.pmed.1003503 (PMC7810318; doi:10.1371/journal.pmed.1003503)
Supplement: Attachment — Submitted filename: Response to reviewers_R2.docx [file pmed.1003503.s006.docx]

30^th^ October 2020

The Editors

PLOS Medicine

Carlyle House

Carlyle Road

Cambridge, CB4 3DN

United Kingdom

Dear Editors,

**RE: Manuscript ID PMEDICINE-D-20-00199R2 entitled “The impact of a routine late third trimester growth scan on the incidence, diagnosis and management of breech presentation in Oxford, UK: A cohort study”**

Thank you very much for returning these reviews to us and for considering our paper.

We enclose our responses to the reviews, gratefully noting that Reviewer 2 and 3 only have positive remarks. We very much hope you will accept this for publication in your journal.

Yours Sincerely

Ibtisam Salim (Corresponding Author) & Lawrence Impey

on behalf of the coauthors.

Dr Ibtisam Salim MD, MMED (Obs/Gyn), MRCOG

Nuffield Department of Women’s and Reproductive Health

Level 3, Womens Centre

John Radcliffe Hospital

Headley Way, Headington

Oxford

OX3 9DU

Email: ibtisam.salim@wrh.ox.ac.uk

Tel: +441865 221004

**A. Requests from the editors:**

1. Bearing in mind the observational design of your study, please adapt wording throughout your paper which ascribes causality to the policy change. For example, at line 44 we ask you to amend the wording to "In this study ... was associated with a reduction in the incidence of undiagnosed breech ...".-
Similarly at line 270, we suggest amending the text to "It is difficult to explain why the policy was associated with a non-significant reduction in emergency CS. While there was an apparent fall in the number of undiagnosed breech babies but not overall ...", or similar*.*

***Authors Response:*** *Thank you. All occasions where causality is ascribed have been altered. This has been predominantly in the discussion. Page 3, Line 44-47 & Page Line 18 239-247*

2. In the title, would "before and after study" be a more appropriate study descriptor than "cohort study"?

***Authors Response:*** *We included the addition ‘a cohort study’ following previous editorial comments. We felt that this and the use of the work impact in the title rendered the use of ‘before and after’ superfluous. We hope this is acceptable to you.*

3. Please harmonize Oxford/Oxfordshire in title and paper. In the abstract, we suggest adapting the text to note that the study was done at a single centre.

***Authors Response:*** *We have done these. Page 2, Line 26.*

4. Please make that "principal" at line 20

***Authors Response:*** *We have done this. Page 2, Line 20.*

5. Please refer to the attached study plan early in the methods section of your main text and highlight any non-prespecified analyses.

***Authors Response:*** *We have done this. The study plan is now a supplementary file. We hope this is what you are requesting. Page 7, Line123.*

*6. Please use the notation "p<0.001" throughout*

***Authors Response:*** *We have done this.*

*7. We suggest removing the attached RCOG abstract.*

***Authors Response:*** *We have done this*

8. In the attached study plan, we suggest deleting the text about the "helper second author".

***Authors Response:*** *We have done this and tidied this up. Please note, as stated in the previous submission, we included this file untouched from its original version so it was transparent the analyses performed were pre specified. It is now included as a supplementary file: we hope this is what is being requested.*

9. Are you able to include participant-level data in supplementary files, or deposit these at a publicly-accessible repository?

***Authors Response:*** *We have uploaded patient-level data as a supplementary file (Excel) as requested. Please note the file size is very large, despite, in this version, having removed all variables that were not used in this analysis.*

**Comments from the reviewers:**

B. Reviewer #2:

The authors have made considerable effort to incorporate the reviewers feedback and comments and the resulting manuscript is clearer and more compelling as a result. The paper present a very large cohort study with interesting findings and I feel it should be published. This iteration of the paper presents a much more clinically-relevant perspective and is much easier to comprehend - the first version was confusing in parts between what was and was not impacted by the US screening intervention. The discussion is now much more balanced and fair, and presents reasonable recommendations for other units based on the findings and limitations of this study.

***Authors Response:*** *We would like to thank Reviewer 2 for his/her time and energy to help us improve this work*

C. Reviewer #3:

Having reviewed the authors' response and the submitted new version of The impact of a routine late third trimester growth scan on the incidence, diagnosis and management of breech presentation in Oxford, UK: A cohort study" (PMEDICINE-D-20-00199R2) by Dr Ibtisam Salim and colleagues for PLOS Medicine I think it is suitable for publication in Plos Med.

Authors responded well to the suggestions of my comments and the second reviewer. The manuscript is clear, only the 2 pre-specified cohorts (pre vs post scan) are used and outcome is well-defined. The new stated conclusion that a routine ultrasound considerably reduced the incidence of undiagnosed term breech presentation at birth is clear, even though this did not result in no undiagnosed breech presentation, a reduction in the incidence of breech presentation and better neonatal outcome. I agree fully with their comment that it is extremely important to show that the introduction of a third trimester scan does not reduce the incidence of breech presentation at delivery, particularly in a unit with widespread and successful use of ECV and therefore obstetricians with clinical skills for vaginal breech delivery should remain.

***Authors Response:*** *We would like to thank Reviewer 3 for his/her time and energy to help us improve this work*

D. Reviewer #4:

I confine my remarks to statistical areas of this paper.  I have a couple of issues to resolve before I recommend publication
1. First, there is no reason to do the tests on lines 137-138.  For "table 1" analysis the key is effect size, not significance. Even very small differences were significant here, but not meaningful. Also, eliminate the p value column in table 1.

***Authors Response:*** *Thank you. Please see answer to point 2. We absolutely accept the significance is less relevant, when the actual differences are very small, but feel that the lack of any meaningful difference between most demographic/ pregnancy groups is a key issue. We wonder if the reviewer meant to eliminate the p values in other tables instead, where there is already a relative risk with 95% CI. We would therefore prefer to keep the lines 137-8 and the p values or at least understand better why examination of the differences might not be considered necessary.*

2. Second, and more importantly, while it isn't exactly *wrong* to do t tests as the authors did, it would be better to use logistic regression and account for covariates. This will increase the statistical power of the tests, and also allow some other conclusions to be drawn.

***Authors Response:*** *Thank you. We had considered this. Please see second half of ‘limitations’ paragraph in the discussion. The covariates for which adjustment might be meaningful would be those that might reasonably have influenced the diagnosis or management of breech presentation, whilst not being on the causal pathway whereby the intervention (a universal scan) is likely to have led to the changes observed. This would mean that adjustment could reasonably be performed for BMI and maternal age, but not number of women having a 36 week growth scan.*

*We did not do this for two reasons. We share Review 4 ‘s views (above) that the small differences observed are not meaningful. More importantly, we are not trying to emulate an RCT: the differences observed reflect the ‘real world’, where BMI and maternal age, at least in developed countries, are increasing. Our findings are perhaps therefore more relevant to examining the ‘impact of introducing a scan’ without such adjustment.*

*We could of course do this but for these reasons would prefer to keep the analysis as it is.*

*We very much hope these responses and changes are acceptable to you.*
