# Supplementary material for: The impact of a routine late third trimester growth scan on the incidence, diagnosis, and management of breech presentation in Oxfordshire, UK: A cohort study
Source: PLoS Med. 2021 Jan 15;18(1):e1003503. doi: 10.1371/journal.pmed.1003503 (PMC7810318; doi:10.1371/journal.pmed.1003503)
Supplement: Attachment — Submitted filename: Response to reviewers_R3.docx [file pmed.1003503.s007.docx]

30^th^ October 2020

The Editors

PLOS Medicine

Carlyle House

Carlyle Road

Cambridge, CB4 3DN

United Kingdom

Dear Editors,

**RE: Manuscript ID PMEDICINE-D-20-00199R3 entitled “The impact of a routine late third trimester growth scan on the incidence, diagnosis and management of breech presentation in Oxford, UK: A cohort study”**

Thank you very much considering accepting our paper for publication.

We enclose our responses to the editors & reviewers. We gratefully note that all reviewers were happy with the manuscript.

Yours Sincerely

Ibtisam Salim (Corresponding Author) & Lawrence Impey

on behalf of the coauthors.

Dr Ibtisam Salim MD, MMED (Obs/Gyn), MRCOG

Nuffield Department of Women’s and Reproductive Health

Level 3, Womens Centre

John Radcliffe Hospital

Headley Way, Headington

Oxford

OX3 9DU

Email: ibtisam.salim@wrh.ox.ac.uk

Tel: +441865 221004

**A: Requests from Editors:**

1. We suggest making that "Oxfordshire" in the title.

***Authors Response:*** *Thank you. We have done this.*

2. At line 26, please adapt the text to "We carried out a population-based ..." or similar.

***Authors Response:*** *Done. Thank you.*

*3.* Please restructure the end of the "Methods and findings" subsection of you abstract. The final sentence should begin "Study limitations include ..." or similar, and should list 2-3 limitations. We suggest that one limitation should be the possible influence of secular changes over time on your findings.

***Authors Response:*** *We have added this.*

4. The final sentence of the "Conclusions" subsection of your abstract seems rather disjointed, and we suggest running on the previous sentence, e.g., "... service, indicating that clinical skills ...". Alternatively the sentence could be removed.

***Authors Response:*** *This has been done.*

5. At line 63, we suggest quoting the actual number of births.

***Authors Response:*** *We have quoted the actual number.*

6. Please remove the quotation marks at lines 63 and 116/7.

***Authors Response:*** *Done. Thank you.*

*7.* At line 72, please amend the text to "... is unlikely to prevent the occasional need for skills ...".

***Authors Response:*** *Done. Thank you.*

8. At line 77, we suggest adapting the text to "... reduced expertise in management of those ...".

***Authors Response:*** *This has been done.*

9. Please begin the sentence at line 239 with "We observed that ..." or similar.

***Authors Response:*** *This has been done. Thank you.*

10. At line 246 and any other instances in the ms, we ask you to amend the word "trend". Please substitute a phrase such as "apparent non-significant benefit".

***Authors Response:*** *This has been done.*

11. At line 323, we suggest substituting "rule out" for "disprove".

***Authors Response:*** *We have substituted the word.*

12. At line 325, please adapt the text to "However, there are also strengths to the study design: ...".

***Authors Response:*** *This has been done. Thank you.*

13. Please remove the information on funding from the acknowledgements section at the end of the main text. This information will appear in the article metadata via entries in the submission form.

***Authors Response:*** *This has been removed. However, I was unable to put the statement below in the submission form. ‘No funding was required for this analysis but the OxGRIP service initiative was jointly funded by Oxford University Hospitals NHS FT and The Oxford Academic Health Sciences Network (AHSN).’*

14. Throughout the text, the labels for tables seem incorrect; e.g., at line 192, "S1_Table" should be "Table 1".

***Authors Response:*** *This has been done.*

15, Please ensure that the attachments (e.g., STROBE checklist) are labelled as they are referred to in the text.

***Authors Response:*** *This is done. Thank you.*

Comments from Reviewers:

**B: Reviewer #3:**

1. The authors have responded to my suggestions.

I suggested removing the p values from Table 1. They said they would like to keep them, or better understand why I asked to remove them. Here is that explanation:

It isn't wrong to put the p values; that is, it doesn't violate any rules or assumptions or whatnot.  But it is misleading. It perpetuates myths about p values. Just as, say, you wouldn't include meaningless medical information (like, say, patient's hair color) so you shouldn't include p values. P values tell you whether to reject a null hypothesis. But, here, there is no null hypothesis. The key issue is whether the two groups are different in any meaningful way. That is answered by the effect sizes. The fact that, e.g. the change in mean maternal age is significant isn't really relevant. It went up by 0.3 years. Unless there is some reason that that difference is of medical significance, why put in the p values?

But I won't insist on the removal, I just think it is a good idea.

***Authors Response:*** *Thank you for clarifying this, and we now understand the point. We have removed the p values on Table 1.*

2. I also suggested using logistic regression instead of t-tests. The authors responded that they felt they did not need to do so because the differences on the covariates were small; they wrote
<<<
The covariates for which adjustment might be meaningful would be those that might reasonably have influenced the diagnosis or management of breech presentation, whilst not being on the causal pathway whereby the intervention (a universal scan) is likely to have led to the changes observed.
>>>

This isn't necessarily so. Even if a variable is equal in the two groups, it can still change the parameter estimates for the main independent variable.  It can also decrease the standard error.

Again, I won't insist on the change because, as I noted originally, it isn't exactly wrong to do t-tests.  But, again, I think logistic reg. would be better.

***Authors Response:*** *Thank you for clarifying this. Our second point, (‘we are not trying to emulate an RCT: the differences observed reflect the ‘real world’, where BMI and maternal age, at least in developed countries, are increasing. Our findings are perhaps therefore more relevant to examining the ‘impact of introducing a scan’ without such adjustment’) remain and we would prefer top leave as it is. Thank you for your understanding.*

**C: Production Issues:**

FIGURES and GRAPHICS:

1. You cite Fig 1 in your manuscript text, though this file is not present in the File Inventory. Please provide this file, or otherwise remove mention of it from your manuscript.

***Authors Response:*** *We have renamed it in the file in the File Inventory.*

2. Please provide all main figures in .tif or .eps format. For more information about how to convert your figure files, please see our guidelines: <http://journals.plos.org/plosbiology/s/figures>. Once you've converted your files to .tif or .eps, please also make sure that your figures meet our format requirements: <http://journals.plos.org/plosbiology/s/figures#loc-file-requirements> / <https://journals.plos.org/plosmedicine/s/figures#loc-how-to-submit-figures-and-captions>

***Authors Response:*** *This has been done. Thank you.*

3. Please ensure that all main figures are referenced in the manuscript as Fig 1, Fig 2, etc. (including capitalization), rather than Figure 1, Figure 2, etc. Please note, however, that the file names themselves must not include the space, i.e. Fig1.tif, Fig2.tif. Please ensure that all main figure files are clearly labeled as such in the File Inventory.

***Authors Response:*** *This has been done. Thank you.*

METADATA:

1. In your Data Availability Statement, please ensure you have provided all necessary access information in complete sentences. This statement will appear on the published version of your manuscript.

**Authors Response:** **Data Availability:** All relevant data are within the manuscript and its Supporting information files.


ARTICLE FILE:

1. Please ensure that your Ethics Statement is available in the Methods section of your manuscript in its entirety, under the subheading "Ethics Statement".

***Authors Response:*** *The Ethics statement is available in the Method section.*

2. We have noted that you have the following present in your manuscript: Abbreviations list

We will remove these section from your manuscript file as this information is automatically pulled from the submission form. Please make sure that the information in the submission form is correct.

***Authors Response:*** *Thank you*

ACKNOWLEDGMENTS:

1. Please remove the funding information from the Acknowledgments section of your manuscript file. The published version is pulled automatically from the “Financial Disclosure” field in our submission form and must not be duplicated. Please therefore take this opportunity to check that the information in this field is correct and completed in full.

***Authors Response:*** *Thank you. This has been done.*

REFERENCES:

1. Please make sure all references are cited in ascending numerical order in the text.

***Authors Response:*** *This has been done.*

SUPPORTING INFORMATION:

1. Please ensure that all Supporting Information files use the following format verbatim throughout the manuscript (including legends and all in-text citations): S1 Fig, S1 Table, S1 Text, S1 Data, etc. Each file type should start at 1 and increase numerically. Please note, however, that the file names themselves must use an underscore rather than a space, i.e. S1_Fig.tif, S1_Table.xlsx, S1_Text.doc, S1_Data.xlsx, S1_STROBE_Checklist.docx, S1_Analysis_Plan.docx, etc.

***Authors Response:*** *This has been done.*

2. Please ensure that each supporting information file has a legend at the end of the manuscript file, after the Reference list.

***Authors Response:*** *This has been done.*

3. Please note that your submission cannot contain any references to generic "supporting information" files and instead must refer to specific files using our naming convention (S1 Fig, S1 Table, S1 Text, S1 Data, etc.)

***Authors Response:*** *This has been done.*

4. If applicable, please replace any page numbers in your STROBE statement with paragraph numbers per section (e.g. "Methods, paragraph 1") since the page numbers of the final published paper may be different from the page numbers in the current manuscript.

***Authors Response:*** *This has been done.*

5. Before resubmitting your paper, please ensure that all files referenced in the manuscript are present in the File Inventory, as missing files will result in further delays. Please also ensure that all file names adhere to our naming conventions and that all in-text citations and supporting information file legends/captions do as well.

***Authors Response:*** *Thank you.* *Files inventory & naming has been checked.*
